# 13,915 reasons for equity in sexual offences legislation: A national school-based survey in South Africa

**DOI:** 10.1186/1475-9276-7-20

**Published:** 2008-07-29

**Authors:** Neil Andersson, Ari Ho-Foster

**Affiliations:** 1 Centro de Investigación de Enfermedades Tropicales, Universidad Autónoma de Guerrero, Apdo 82, Acapulco, México; 2 CIET Trust, 71 Oxford Road, Saxonwold, Johannesburg, 2196, South Africa

## Abstract

**Objective:**

Prior to 2007, forced sex with male children in South Africa did not count as rape but as "indecent assault", a much less serious offence. This study sought to document prevalence of male sexual violence among school-going youth.

**Design:**

A facilitated self-administered questionnaire in nine of the 11 official languages in a stratified (province/metro/urban/rural) last stage random national sample.

**Setting:**

Teams visited 5162 classes in 1191 schools, in October and November 2002.

**Participants:**

A total of 269,705 learners aged 10–19 years in grades 6–11. Of these, 126,696 were male.

**Main outcome measures:**

Schoolchildren answered questions about exposure in the last year to insults, beating, unwanted touching and forced sex. They indicated the sex of the perpetrator, and whether this was a family member, a fellow schoolchild, a teacher or another adult. Respondents also gave the age when they first suffered forced sex and when they first had consensual sex.

**Results:**

Some 9% (weighted value based on 13915/127097) of male respondents aged 11–19 years reported forced sex in the last year. Of those aged 18 years at the time of the survey, 44% (weighted value of 5385/11450) said they had been forced to have sex in their lives and 50% reported consensual sex. Perpetrators were most frequently an adult not from their own family, followed closely in frequency by other schoolchildren. Some 32% said the perpetrator was male, 41% said she was female and 27% said they had been forced to have sex by both male and female perpetrators. Male abuse of schoolboys was more common in rural areas while female perpetration was more an urban phenomenon.

**Conclusion:**

This study uncovers endemic sexual abuse of male children that was suspected but hitherto only poorly documented. Legal recognition of the criminality of rape of male children is a first step. The next steps include serious investment in supporting male victims of abuse, and in prevention of all childhood sexual abuse.

## Introduction

In May 2007, the Sexual Offences Bill passed by the South African National Assembly expanded the definition of rape to include forced sex with men [1]. Until then, sexual abuse of boy children could only be charged as indecent assault – a considerably more trivial offence than rape.

Each year in South Africa, there are over 50,000 rapes and attempted rapes reported to the South African Police Services – and less than 10,000 indecent assaults. Apart from rape being a more serious offence, the implication here is that at least five times more females are raped than males. Sexual abuse of female victims can also be charged as indecent assault.

Recent publicity of sexual abuse in church schools, and sizeable compensation paid by the Catholic Church to victims of abuse in Canada and the USA, has focused international attention on male victims of sexual abuse. Recent reports of male abuse among peers at schools in the USA and Canada [2-4], Germany [5], Ghana [6] and Zimbabwe [7] indicate that, if looked for, abuse of boy children is fairly common. Female perpetrators are also beginning to receive attention internationally [8].

We set out to find out how common male sexual abuse is in South Africa, and to identify some of the relationships between male child rape and behaviours of schoolboys.

## Methods

### Sample

Based on census data, we divided electoral areas into metro/capital, urban and rural. Proportional to the population in each resulting stratum, we drew a random sample of enumeration areas. From a list of all registered schools, we matched schools to each enumeration area, identifying a total of 1194 schools in this way. In three of the nine provinces, additional funding allowed over-sampling to increase the local relevance.

### Ethical review

The Provincial Departments of Education in all nine provinces gave permission for the study as part of curricular activity, typically in the context of Life Skills classes. The facilitator explained to each class that the questionnaire was voluntary and could be stopped at any time. Facilitators also explained that no questionnaire would be marked with an identity, and they arranged classroom logistics to permit each learner some privacy.

### Concept development and pilot

Because there is no word for rape in several of the South African languages, we used the expression "forced sex without consent" in nine of the 11 official languages. We arrived at this through feedback from results of a pilot study that included 9000 youth in urban, rural and remote communities in the nine provinces (27 pilot sites, in nine languages). The pilot questionnaire used "rape" or its equivalent in three languages, and a variety of phrases in other languages, intended to communicate the same meaning. In each site, separate focus groups for male and female youth considered the pilot results and discussed the wording in their own language. After translation and back-translation of the resulting phrase by someone not associated with the study, the final formulation went through two to five rounds of questionnaire piloting in the nine languages of implementation. Focus groups in urban and rural areas in each province discussed and validated the outcomes, as the design team tried to be sure we were measuring what we intended to measure.

### Instruments

The anonymous, facilitated self-administered instrument included questions on attitudes and experience regarding sexual violence and HIV risk. In each classroom, a facilitator read each question and explained its meaning following a pre-tested script in English, Sesotho, Sepedi, Setswana, Setsonga, Tshivenda, IsiZulu, IsiXhosa and Afrikaans, depending on the needs of the class.

### Outcomes

Schoolchildren answered questions about the following outcome measures: did you suffer forced sex without consent in the last year; have you ever been forced to have sex without your consent by a learner, a teacher, another adult, a family member; at what age were you first forced to have sex without your consent; were you forced to have sex without your consent by a male, female, both. In addition to their age and sex, learners also provide information on HIV risk-related knowledge, attitudes and practices, their exposure and preferences towards national intervention programmes, and perceived HIV status – we share findings concerning those measures elsewhere [9].

### Data collection and management

Data collection took place from 7 October to 22 November 2002. Teams visited a total of 5162 classes in 1191 schools. We employed several measures to reduce bias. Facilitators asked educators to leave the class prior to the survey, and asked participants not to write their names or any identifying marks on the questionnaires. Facilitators made serious efforts to prevent viewing of questionnaire responses by nearby students, instructing children to cover questionnaire responses with exercise books. They arranged for the provision of "shield" books for pupils who did not have one. Respondents completed questionnaires on their own, turning them facedown once completed. Facilitators collected questionnaires from learners and placed them in an envelope which they immediately sealed. The sealed envelopes were only opened again at data entry. We informed learners of this process prior to handing out questionnaires, to assure them their responses would remain anonymous. Four scanners read and verified data from the questionnaires.

### Analysis

We rebalanced unequal representation of provinces by weighting estimates of national occurrence indicators of forced sex in the last year and "ever". The full sample and the raising factors applied to estimate national prevalence rates are reflected elsewhere [9]. Risk analysis used the Mantel-Haenszel procedure [10] which stratifies the main contrast by other factors to make sure the finding cannot be explained by covariants (age, sex, HIV risk-related knowledge, attitudes and practices, exposure and preferences towards national intervention programmes, and perceived HIV status).

We adjusted for the dependency between reports from participants from the same cluster, using the adjusted Mantel-Haenszel chi-square statistics of Zhang and Boos [11]. This reduces chi-square estimate, increasing the confidence intervals roughly in proportion to the intra-cluster correlation coefficient. We opted for 99% confidence intervals to offset the effect of multiple testing in the principal contrasts. We then examined the mutual influence of factors that affected forced sex using logistic regression (stepping down from a saturated model) using CIETmap, which derives odds ratios for each determinant, taking into account the others in the final model [12]. The saturated initial model included urban/rural, type of school, province, age, attitudes about sex (need to have sex to show love, girls have the right to refuse sex, girls like sexually violent guys), age at sexual debut, how often they talk about sex, ever forced sex with someone else, believe condoms prevent HIV/AIDS, belief about personal HIV status and other abuse (verbal, beating).

## Findings

### Occurrence of male child rape

Weighted by province and urban/rural areas, 9% (based on 13915/127097) reported forced sex without consent in the last year. In answer to a separate question, 44% of 18 year-olds said they had "ever" been forced to have sex (weighted value of 5385/11450).

#### Age

The age of 126,696 male respondents ranged from 10 years to 19 years (average age 15 years, SD 1.426). Reports of forced sex in the last year varied across age groups: 14% (87/614) at age 10 years, 10% (436/4353) at age 11, 9.8% (1253/12729) at 12 years, 9.5% (1643/17251) at 13 years; 10.4% (2029/19,536) at 14; 11% (2126/19337) at 15 years; 11% at 16 years (2049/18711); 11.9% (1886/15890) at 17 years; 12.8% (1467/11450) at 18 years and 13% (893/6825) at 19 years.

#### Urban/rural

Rural schoolboys were more likely than their urban or metro counterparts to report forced sex in the last year (odds ratio 1.7, 99%CI 1.42–1.99; 9659/74382 reported forced sex in rural areas compared with 4256/52715 in urban areas).

#### Province

There was also a notable difference between provinces, with Limpopo (the least economically developed and mostly rural province) suffering the highest rates and Western Cape the lowest. Table 1 shows the rates of forced sex in the last year across the nine provinces.

**Table 1:** Percent (and number affected) of schoolboys aged 10–18 years who reported being forced to have sex

	Provincial characteristics	Male youth forced to have sex in the last year % (number raped)
Limpopo	Most northerly province, four major ethnic groups, least economically developed, mining provides its main income; 10% of the land, 12% of the national population; 77% living in poverty – 6.6 assaults reported per 1000 people per year	16.1% (n7653)
Mpumalanga	Agriculture, tourism, manufacturing and mining; 6% of the land, 7% of the national population; 57% living in poverty – 8.6 assaults reported per 1000 people per year	11.9% (n302)
Northwest	Mining and agriculture; 9% of the land, 8% of the national population; 52% living in poverty – 10 assaults reported per 1000 people per year	10.6% (n349)
Free State	Economy based on mining and agriculture; 11% of the land, 6% of the national population; 68% living in poverty – 13.7 assaults reported per 1000 people per year	10.3% (n276)
Northern Cape	A new province since 1994, diamonds provide main income; 31% of the land, 2% of the national population; 61% living in poverty – 25 assaults reported per 1000 people per year	10.0% (n182)
Gauteng	Economy based on mining, finance, manufacturing; highest income/capita, highest literacy, highest population density (576 per sq km); 1% of the land, 19% of the national population; 42% living in poverty – 11.2 assaults reported per 1000 people per year	8.3% (n528)
KwaZulu Natal	Economy based on tourism and agriculture; 8% of the land, 21% of the national population; 61% living in poverty – 6 assaults reported per 1000 people per year	7.7% (n3354)
Eastern Cape	Economy based on agriculture, livestock and manufacturing; 14% of the land, 14% of the national population; 72% living in poverty – 10 assaults reported per 1000 people per year	6.9% (n1162)
Western Cape	Highly developed, diverse economy; 11% of the land, 10% of the national population; 32% living in poverty – 16.5 assaults reported per 1000 people per year	4.8% (n109)

in the year prior to the survey.

Sources of information on provinces ;

#### Beating and other abuse

Weighted by province and urban/rural, 21% (29,296/127,097) of schoolboys reported verbal insults in the last year; 15% (22768/122666) reported being beaten in the last year; 15% (22525/127097) reported unwanted touching. There was a marked association between schoolboys who had been beaten and those forced to have sex in the last year (odds ratio 4.17, 99%CI 3.1–5.18; 5923/22768 of those who had been beaten were also forced to have sex, compared with 7509/99898 of those who had not been beaten in the last year). In urban areas, the association between beating and forced sex was slightly stronger (OR 4.96, 99%CI 3.9–6.01, 1740/7826 forced among those who had been beaten 2352/43174 forced among those not beaten) than in rural areas (OR 3.89, 99%CI 2.98–5.1; 4143/14942 forced among those who had been beaten compared with 5157/56724 forced among those not beaten).

### Age of first consensual and forced sex

**Figure 1:**
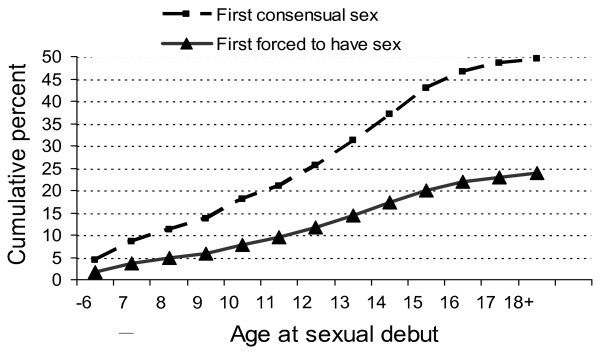
**Cumulative rates of forced and consensual sex, among South African schoolboys who reported an age of sexual debut**.

Some 20% (25,698/127,097) of all male respondents gave an age when they were first forced to have sex (some may have been forced many times). We used this as the basis for exploring age related patterns. Excluding the 19,271 schoolboys who said they were raped in the last year or ever, but did not give an age when this first occurred, Figure 1 shows the cumulative first rape between the ages of six and 18 years, based on 1919, 1921, 1358, 1174, 2183, 1554, 2583, 2794, 3045, 3131, 1964, 1179 and 893 reports each year between the ages of 6 or less, and 18. It also shows the cumulative age of first consensual sex, based on 5479, 5273, 3584, 3016, 5639, 3630, 6106, 6969, 7412, 7450, 4638, 2439 and 1290 reports each year between the ages of 6 or less, and 18.

### The perpetrators

Some 28% (12661/44969) of those who had been abused said they were forced by an adult (not family or a teacher). Another 28% (12578/44969) said they had been forced to have sex by a fellow schoolchild, while 20% had been abused by a teacher (9038/44969) and18% (7985/44969) by an adult family member. An important proportion reported being forced to have sex by more than one type of perpetrator: 25% of schoolboys (11040/44969) were victims of sexual abuse by at least two types of perpetrator (schoolchild, teacher, family member or other adult). Some 8% (3595/44969) reported being forced to have sex by both fellow students and teachers.

Rural respondents were significantly more likely to report abuse at school by a fellow student and teacher than were urban counterparts (odds ratio 1.36, 95%CI 1.25–1.47; 2288/29836 rural abused said they had been raped by *both *students and teachers, compared with 874/15153 urban schoolboys who had been abused).

In response to the question about the sex of the perpetrator, 32% of those who answered (7755/23889) said the perpetrator was male, 41% (weighted value for 9879/23889) said she was female and 26% (6255/23889) said they had been forced to have sex by both male and female perpetrators. Male abuse of schoolboys was much more common in rural areas while female perpetration was more an urban phenomenon. Excluding those who had been abused by both male and females (which was not very different a proportion in urban and rural areas), an urban schoolboy was less like to be abused by a male than was his rural counterpart (odds ratio 0.67, 95%CI 0.92–0.47; 2125/5677 urban victims were abused by males compared with 5630/11957 rural victims).

There was also an important association between victim age and sex of the perpetrator. Again excluding victims who had suffered abuse from both male and female perpetrators, younger victims (aged 10–14 years at the time of the enquiry) were more likely to report a male perpetrator than those aged 15–19 years (odds ratio 1.65, 95%CI 1.26–2.06; 3331/6424 younger victims reported a male perpetrator, compared with 4391/11137 older victims).

### Teachers soliciting sex

One in every twenty schoolboys (4.6%, weighted value of 7125/121491) said they had been asked to have sex by a teacher. This was significantly less common in urban than rural areas (odds ratio 0.52 95%CI 0.71–0.33; 1981/50716 in urban areas and 5144/70775 in rural areas were asked by a teacher to have sex). Reports of teacher solicitation increased steadily with age (145/4181, 491/12243, 703/16554, 916/18749, 1026/18537, 1131/17888, 1131/15145, 940/10838 and 605/6438 for 11 to 19 years of age respectively.)

### Victims become villains

Some 11% (13977/127097) of male respondents said they had forced sex on someone else. This report was more common in rural than urban areas (8762/74382 in rural compared with 5215/52715 in urban areas said they had forced sex on someone else; this difference was not statistically significant after taking account of the effect of clustering). Youth perpetration of sexual violence was marginally more common in poorly resourced schools (7303/66287 in poorly resourced schools compared with 3284/34840 in well resourced schools, although this difference was not statistically significant after taking account of the effect of clustering).

## Discussion

Male schoolchildren in South Africa suffer high rates of sexual abuse, many of the assaults perpetrated in school. By the age of 18 years, two in every five schoolboys reported being forced to have sex, mostly by female perpetrators. These rates of sexual violence are consistent with at least one other national study [13].

In this national sample, suffering forced sex was associated with a history of beating and verbal insults. Younger males were more likely to be abused by male perpetrators. Male perpetrators were more common in rural areas while female perpetrators were more commonly reported in urban areas. One in ten school boys surveyed admitted they had forced sex on someone else.

The considerable size and national representation of this survey under controlled classroom conditions provides unprecedented power to estimate rates of sexual abuse. The survey instrument did not allow for more nuance or discussion of responses. Another regrettable limitation is that we cannot estimate the overall *burden *of sexual abuse: how many times each child was forced to have sex or the degree of accompanying violence. We did not distinguish transactional sex, although it is not strictly forced sex without consent. And we did not document the age of the perpetrator when this was a fellow school child.

A general limitation of all questionnaire-based research on sexual violence is that the information depends entirely on the response of the participant. They can exaggerate and they can withhold information, and we have no way to verify this. It remains a weakness of all questionnaire-based enquiries of sexual violence. The instrument development involved a rigorous design process where we validated questionnaire responses through qualitative follow up in gender-stratified focus groups in each pilot site. Translation and back translation processes relied on language speakers from areas similar to the study population.

This study was a cross-section of children present at sample schools during a single field visit. The anonymous, facilitated self-administered questionnaire prevented registering class members not present at the time of the visit, and no effort was made to contact those who were not present as this would make them identifiable as individuals. It seems reasonable to assume that, if anything, the survey underestimated sexual violence among schoolboys.

### Potential policy implications

Boys who are victimized quite probably experience a similar range of psychological consequences as girls. Studies of adolescent males have also found an association between suffering rape and a variety of negative behaviors including absenteeism from school [14]. Boys who are perpetrators of gender violence can also be viewed as victims of a narrowly constructed male gender role that provides boys limited opportunities for expressing their masculinity and condones or even encourages displays of power over girls as appropriate behaviour. It seems reasonable to expect similar power dynamics will affect both sexes.

Many child perpetrators of rape have themselves been victims of sexual abuse [15]. It is also well established that people who have been sexually abused as children are more likely to become abusers themselves [16-18]. There is increasing recognition of links between sexual abuse and high-risk attitudes to sexual violence and HIV risk [19-21]; sexually abused children are also more likely to engage in HIV high-risk behaviour [22].

The likely consequence of all this for South African society is the multiplication of sexual abuse. Our findings offer strong support to the 2007 Sexual Offences Bill, indicate the need to raise awareness about rape of male children, and warrant further efforts to prevent sexual violence in South Africa.

## Authors' contributions

AHF participated in the design of the study and supported the statistical analysis. NA conceived of the study, participated in its design and coordination, and conducted the principal analysis. Both authors read and approved the final manuscript.
